# Impact of the COVID-19 Pandemic on the Emotional Health of Children Under 6 Years in Washington, DC †

**DOI:** 10.3390/children12080981

**Published:** 2025-07-26

**Authors:** Tom Kariyil, Miranda Gabriel, Kavya Sanghavi, Elizabeth M. Chawla

**Affiliations:** 1 School of Medicine, Georgetown University, Washington, DC 20007, USA; tjk92@georgetown.edu; 2 College of Arts & Sciences, Georgetown University, Washington, DC 20007, USA; mcg143@georgetown.edu; 3 Medstar Health Research Institute, Columbia, MD 21044, USA; kavya.k.sanghavi@medstar.net; 4 Department of Pediatrics, Medstar Georgetown University Hospital, 4200 Wisconsin Ave NW, Suite 200, Washington, DC 20016, USA

**Keywords:** early-life stress, emotional health, COVID-19 pandemic, infant and early childhood mental health, parenting, pediatric primary care

## Abstract

**Background/Objective:** A growing body of international research continues to show evidence of worsening youth mental health since the beginning of the COVID-19 global pandemic, yet very little research in this area has included young children under 6 years. Given the potential impact of early life stress during this critical period of development, it is crucial to better understand the effects on this age group. The objective of this study was to better understand the impact of the COVID-19 pandemic on the emotional health of very young children. **Methods:** This study utilized retrospective chart review of primary care records to compare the prevalence of markers of stress in two cohorts of children under the age of 6 years, comparing children presenting for care prior to the pandemic (1 April 2019–31 March 2020; control period) with those presenting for care during the first year of the pandemic (1 April 2020–31 March 2021; study period) in a large pediatric primary care clinic in Washington, DC, USA. Based on power calculations, charts of 200 patients from each cohort were reviewed and prevalence of stress markers were summarized using counts and percentages and compared between groups using chi-squared tests. Multivariable logistic regression models were also conducted for each domain adjusting for age, gender, and insurance type. **Results:** Overall, sleep difficulties were significantly more prevalent during the pandemic period compared to the control period (14% vs. 6.5%, *p* = 0.013). In addition, signs of stress presented differently across age groups. For example, during the pandemic period toddlers (13–35 months) were 13 times more likely (OR = 13, 95% CI [2.82, 60.4], *p* < 0.001) and preschool-aged children (36–71 months) were 18.5 times more likely (OR = 18.5, 95% CI [4.0, 86], *p* < 0.001) than infants to present with behavior problems, indicating substantially higher risk of externalizing symptoms in older children compared to infants. Toddlers were less likely than infants to present with mood changes (e.g., fussiness or crying) (OR = 0.15, 95% CI [0.03, 0.65], *p* = 0.011). In addition, toddlers (OR = 0.55, 95% CI [0.31, 0.97], *p* = 0.038) and preschool-aged children (OR = 0.15, 95% CI [0.06, 0.4], *p* < 0.001) were also less likely to present with feeding difficulties compared to infants. **Conclusions:** One of the very few studies of young children under 6 years (including infants) during the COVID-19 pandemic, this study found that even very young children experienced stress during the pandemic. Signs of emotional stress were identified in a primary care office during routine care, highlighting an important opportunity for early intervention and/or prevention, such as counseling and resources for caregivers, in settings where young children are already presenting for routine care.

## 1. Introduction

The COVID-19 global pandemic, as well as responses to address and mitigate the spread of this disease, led to significant changes in the lives and daily routines of many children and families around the world. A growing body of literature continues to show this period of change has had a significant impact on the mental and emotional health of children globally [1,2,3,4,5,6,7,8,9,10]. A systematic review of 21 studies across 11 countries published in 2023 found overall evidence of a “longitudinal deterioration in the mental health” of children and adolescents, with increases in anxiety, depression, and psychological distress after the start of the pandemic [1]. In the United States, research has shown an increase in eating disorders and mood disorders in youth [2,3], as well as increases in suicidality in adolescents since the beginning of the pandemic [11], which led to a joint statement of a “youth mental health crisis” in the United States in 2022 [12].

Despite this growing body of research of youth mental health, few studies have included children under the age of 6 years, and even fewer studies have examined the impact of the global pandemic on the emotional health of very young children and infants. Of the 21 studies included in the systematic review, only 5 included children below the age of 10 years, and none included children under 3 years [1]. Of the very few published studies including children under 2 years [6], to our knowledge none have included infants. Children under 3 years remain an understudied population in relation to the emotional health impacts of the global pandemic.

However, early childhood is a critical time for the development of the growing brain, as well as foundations of emotional health such as secure attachment, problem solving, and self-regulation [13]. Changes to families and routines associated with the COVID-19 pandemic could potentially impact core needs of infant mental health including secure relationships, supportive co-regulation from a caregiver, and safe environments for developmental learning [14]. Not surprisingly, recent studies of maternal mental health have shown associations between maternal depression and worsening of children’s mental health and even achievement of developmental milestones [8,15]. Stress or disruptions to the normal development of emotional health in this period could potentially impact children for years to come and thus is an important area of study.

Limited existing research of young children during the pandemic has shown increases in mental/behavioral health symptoms specific to this age group, most notably increases in behavior problems in preschool-aged children [5,6,7,8,9,10]. However, many of these studies either relied on surveys of parental report of child behaviors and symptoms [4,5,6,7,8,9,10], which could be influenced by parental perception of behaviors, or only included children who had already presented for mental health services [8,16]. Yet some studies have suggested externalizing symptoms such as behavior problems were more likely to be reported by parents or lead to families seeking mental health services during the pandemic [7,16], whereas young children with internalizing symptoms or more subtle signs of stress may have been less apparent [14].

In response to the global decline in child mental health in the years following the pandemic, the WHO and UNICEF released new guidelines in 2024 for child and adolescent mental health screening, support, and resource development [17]. Recognizing the lack of providers and difficulty accessing mental health services in many countries throughout the world, the WHO emphasized the need to improve mental health care delivery in settings where youth already access care and the need to develop skills in providers already embedded in communities such as general practitioners and community health teams. Not only does embedding mental health surveillance and care delivery into primary care settings offer the opportunity to increase the number of children and families that can be reached, it also offers an opportunity to identify problems at an earlier stage which is critical for prevention and early-intervention strategies.

The aim of this study was to examine the impact of the global pandemic on the emotional health of children under 6 years presenting for routine care within the primary care setting during the COVID-19 pandemic. We hypothesized that: (1) young children under 6 years, even as young as infants, would show increased stress markers during the pandemic period as compared to pre-pandemic, (2) very young children experiencing stress are more likely to present with more subtle signs than older children which may limit their identification, and (3) evaluating young children for signs of stress in the primary care setting could illuminate a broader spectrum of affected children than specialty mental health services. Although relatively little is known about the impact of the pandemic on the emotional health of children under 6 years, identifying stress in young children during this critical period of development could offer insights with potentially significant impacts.

## 2. Materials and Methods

### 2.1. Study Design and Study Population

This retrospective study utilized chart reviews of two cohorts of children under 6 years presenting for routine care in a primary care clinic within the Medstar Georgetown University Hospital system. This clinic cares for patients from the greater Washington DC area, as well as from the surrounding states of Maryland and Virginia, all of which had varying degrees of quarantine during the first year of the pandemic. Cohort one included children presenting for care prior to the pandemic (1 April 2019–31 March 2020; control period), and cohort two included children presenting for care during the first year of the pandemic (1 April 2020–31 March 2021; study period). We chose the first year of the COVID-19 pandemic as our study period specifically because this was the time with the most significant changes to daily life in our geographic area, such as at-home quarantine, closure of schools and daycare centers, etc., to best capture impacts most directly related to the pandemic itself in our population.

Inclusion criteria consisted of patients aged 0–71 months at first visit during a specific time period (study or control), those who were seen for routine care at least once and had complete provider documentation for all visit(s) during the specific period. Exclusion criteria included any patient not seen during the respective period, not seen for routine care (e.g., only subspecialist or emergency care encounters), or patients who had missing or incomplete documentation for any visit during the indicated time period. Children from all races, ethnicities, nationalities, immigration status, insurance status, primary language, and family structure (e.g., single parent, two parents, same-sex parents, foster children) were included.

Using billing records limited to patient visits within primary care for children under 6 years of age at the start of the cohort period, an initial data set of approximately 9000 patient visits per study period was generated. To achieve 80% power to detect a 15% difference, 200 discrete patients were randomly selected in each study group from this data set using a random number generator. Power calculations were based on pre-pandemic prevalence data in the literature, which was quite variable across our domains of interest [18,19,20]. Despite this variability, we chose a baseline prevalence of 20% for at least one domain for our calculations, which was most consistent with existing literature for the majority of our domains of interest. We chose to power the study to detect a 15% percentage point difference, as we anticipated a prevalence rate of at least 35% for one or more domain based on early studies of children during the pandemic [4,6].

### 2.2. Data Collection and Outcomes

Primary outcomes included measures of emotional distress in young children. We measured the prevalence of distress by counting the presence (yes/no) of a concern or change in one of five domains (sleep, behavior, mood, feeding, and toileting) as expressed by the caregiver during the encounter, which are consistent with signs of stress in this age group in the literature [21,22]. Several published scales such as the Pediatric Emotional Distress Scale (PEDS) are not validated under 2 years of age, and while the ASQ-SE does include infants this questionnaire is lengthy and not currently part of our patient care workflow. As the study aim was to evaluate young children for signs of stress during routine care, we opted not to use one of these existing questionnaires. Of note, discussion of the five domains is included as part of routine care for young children in the United States through the American Academy of Pediatrics “Bright Futures Guidelines” [23], and therefore consistently discussed during visits and present in routine documentation.

Patient demographics, visit details, and visit documentation limited to the specified time period were initially extracted by one study team member (EC), then two additional study team members blinded to cohort (TK and MG) reviewed all primary care notes and documentation for primary outcome measures for all patients after several pilot reviews for training and alignment between reviewers. All patient demographics were blinded to reviewers as they only received the body of the visit documentation, and patients from each cohort were randomly mixed for review.

### 2.3. Data Analysis

Patient characteristics and prevalence of concern in any domain were summarized using counts and percentages and compared between groups using chi-squared tests. Differences between groups stratified by age were also analyzed, including infants (0–12 months), toddlers (13–35 months), and preschool-aged children (36–71 months). In addition, multivariable logistic regression models were conducted for each domain adjusting for age, gender, and insurance type. Children from diverse backgrounds (e.g., ethnicity, nationality, language) and family structures were included in all groups. We did not adjust for each of these factors individually, with the exception of insurance type which served as a surrogate marker of socio-economic status. All analyses were conducted in Stata 17, and statistical significance was defined by *p*-values < 0.05.

## 3. Results

A total of 200 patients in each cohort were included for analysis. Patients in each group were similar in age ($\bar{x}$ = 17 months [control] vs. $\bar{x}$ = 18.75 months [study]), gender, and type of initial encounter (i.e., new or returning patient), but there was a significant difference in type of insurance and type of visit (i.e., in-person or tele-health visit) between study groups (Table 1).

**Figure 1:**
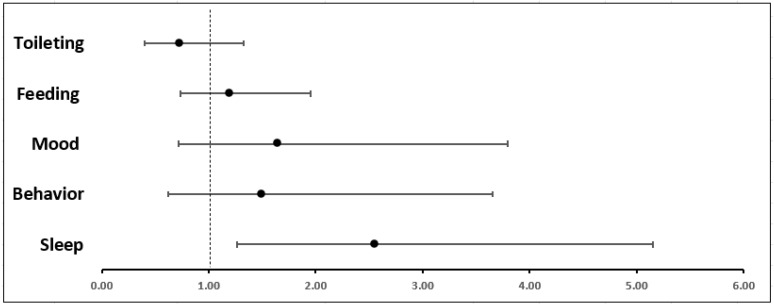
Adjusted odd ratios: Odds of presenting with signs of stress during study period in each domain, adjusted for gender, age, and insurance type.

Signs of stress presented in all five domains in both groups (Table 2). However, sleep difficulties were more prevalent during the COVID-19 pandemic for all age groups (14% vs. 6.5%, *p* = 0.013). Concerns in all other domains were more prevalent during the study period, apart from changes in toileting, but did not reach significance. This held true even when adjusted for age, gender, and insurance type (Figure 1), as children of all ages were 2.56 times more likely to present with sleep difficulties during the pandemic period as compared to the control period (OR = 2.56, 95% CI [1.27, 5.15], *p* = 0.009).

### 3.1. Age Associated Differences

**Figure 2:**
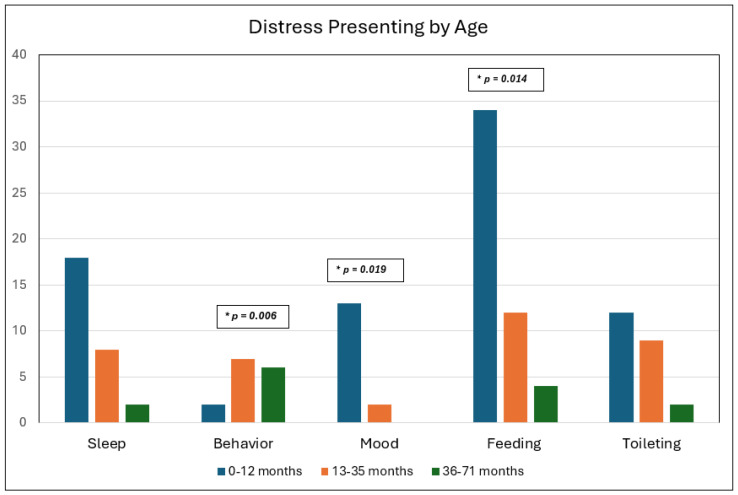
Differences in Presentation of Stress During the Study Period Stratified by Age Group.

Although concerns in all five domains were present during the study period, there were differences in how stress presented within different age groups. Stratified by age, mood changes/fussiness (*p* = 0.019) and feeding difficulties (*p* = 0.014) were more prevalent in infants (0–12 months), whereas toddlers (13–35 months) and preschool-aged children (36–71 months) were more likely to present with behavior concerns (*p* = 0.006) (Figure 2).

**Figure 3:**
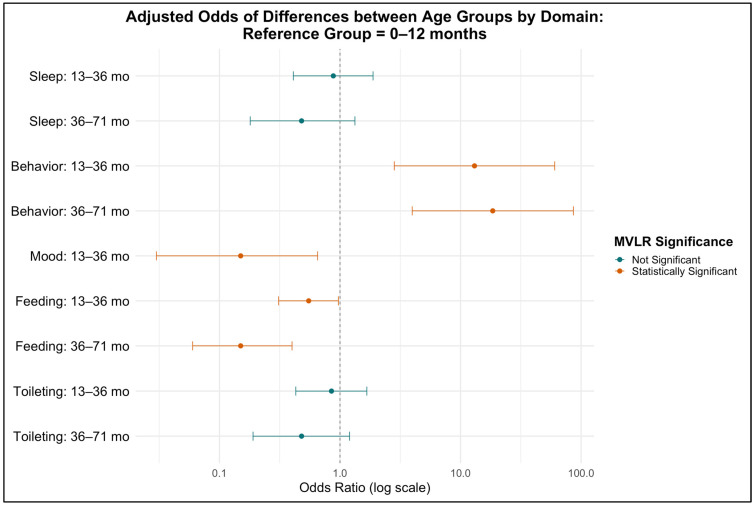
Multivariable logistic regression: Odds of stress symptoms presenting in different age groups [reference group infants], adjusted for gender, and insurance type.

These age-related differences remained true even when adjusting for other variables: multivariable logistic regression models were conducted for each domain, adjusting for age, gender, and insurance type (Table 3). This analysis also confirmed that certain signs of stress were more common in specific age groups. For example, toddlers were 13 times more likely (OR = 13, 95% CI [2.82, 60.4], *p* < 0.001) and preschool-aged children were 18.5 times more likely (OR = 18.5, 95% CI [4.0, 86], *p* < 0.001) than infants to present with externalizing symptoms such as behavior problems. However, toddlers were less likely than infants to present with mood changes (e.g., fussiness or crying) (OR = 0.15, 95% CI [0.03, 0.65], *p* = 0.011). In addition, toddlers (OR = 0.55, 95% CI [0.31, 0.97], *p* = 0.038) and preschool-aged children (OR = 0.15, 95% CI [0.06, 0.4], *p* < 0.001) were also less likely to present with feeding difficulties as compared to infants (Figure 3).

### 3.2. Differences Within Groups

Although the total number of patients presenting with signs of stress was not statistically significant between groups, more patients in the study period presented with two or more positive domains indicating certain individuals had much higher levels of stress during the pandemic. Of those patients with any sign of stress, 29% (n = 23) in the control period had more than one positive domain whereas in the study group 42.5% (n = 34) had more than one positive domain, which approached but did not reach significance (*p* = 0.078). In addition, during the study period those patients on public insurance were 2.62 times more likely to be referred to a specialist (e.g., behavior specialist) for the concern as compared to those on private insurance (OR = 2.62, 95% CI [1.19, 5.81], *p* = 0.017).

Not all young children experiencing distress during the study period presented to care specifically for that reason, as many were identified during routine primary or preventive care. Of those patients experiencing some form of stress, only about half (56%, *n* = 45) initiated a visit with the provider for that concern (as described in the chief complaint). For almost half of patients (44%, *n* = 35) experiencing early childhood stress during the study period, this was identified during a routine care visit.

## 4. Discussion

One of the very few studies of children under 6 years, the purpose of this study was to investigate the impact of the COVID-19 pandemic on the emotional health of children under 6 years presenting for routine care. We found evidence of increased stress during the pandemic period in this age group, even in infants under 12 months. Significant findings included increases in sleep difficulties during the study period for all ages and subgroups. This finding is consistent with other studies from the pandemic of young children, older children and even caregivers of young children [4,5,6,10]. A recent meta-analysis found possibly up to 54% of children and adolescents experienced sleep disturbance during the pandemic, although authors found wide variability in measures and study design among the studies in this review [24]. However additional studies from the pandemic period in communities around the world continue to show sleep was often affected during the pandemic for all age groups, so it is not surprising this would include children under 6 years. Poor sleep in early childhood can have impacts on behavioral and developmental milestones, as well as direct impacts on emotional regulation [5,25,26]. Perhaps related, we also found increases in behavior problems in toddlers and preschool-aged children, which is consistent with studies of preschool-aged children from the pandemic [5,6,7,9,10]. Within the study period, stress presented differently across different age groups: feeding difficulties and mood changes were more common in infants while behavior problems were more common in toddlers and preschool-aged children.

We noted an increase in the prevalence of stress in all domains during the study period; however, these increases did not reach significance except in the domain of sleep. This could be because our sample size was not large enough to reach significance. Power calculations were based on pre-pandemic prevalence data in the literature, which was quite variable across our domains of interest. For example, pre-pandemic studies of sleep problems in children ranged from 10 to 30% [18], behavior problems ranged from 15 to 20% [19], and limited studies indicate likely 10 to 20% of infants experience increased fussiness [20]. In addition, as studies of children under 6 are often lacking, some of these existing studies were based on slightly older cohorts of children and therefore may not have been representative of prevalence in our target population. Despite this variability, we chose a baseline prevalence of 20% for at least one domain for our power calculations. However, prevalence rates in our control group were lower than published baseline prevalence for most domains, and far lower than 20% for several of our domains. This could be related to the difficulty in measuring these stress domains in this age group, and/or our study design which focused on identifying signs of stress as it presented in discussion during routine care and not asking caregivers directly through questionnaires. In addition, several of our significant findings had wide confidence intervals, which could suggest the need for larger sample sizes. Although overall this study shows smaller increases in stress during COVID-19 than studies of older children, the findings have importance given the potential long-term impacts of stress during this crucial period of development.

At the individual level, we found higher levels of stress during the pandemic for some families. Although the total number of patients presenting with any sign of stress was not statistically significant between groups, more patients in the study period presented with two or more positive domains indicating higher levels of stress for certain individuals during the pandemic.

In addition, more children were on public insurance during the pandemic period in our study population. In the United States, public insurance is only available to families who fall below a maximum income level (or percentage of the ‘poverty line’), or less commonly meet certain other criteria such as complex medical problems. There were no differences between study groups in terms of new patients (36% vs. 34%, *p =* 0.675) indicating the study period likely did not include a significant number of new patients to our health system and our two groups likely represented a similar cohort of patients. It is more likely that our existing patient panel had an increase in the number of patients that qualified for public insurance during the pandemic period, which could indicate loss of employment-based private insurance or loss of income that moved them into a qualification bracket. This is consistent with the literature showing loss of income and employment during the first year of the pandemic [27]. Our study also found that children on public insurance were more likely to be referred to a specialist during the study period, which could indicate higher levels of distress and/or a greater impact on family functioning and therefore need for external help. This is consistent with studies from other countries which found families with greater economic challenges during the pandemic had higher levels of stress [7,8].

We also found differences in the presentation of stress across different age groups, which is congruent with developmental stages and consistent with other studies showing differences in mental health concerns across age groups during the pandemic [6]. However, this is one of the first studies to include infants under one year of age. Our study found that even infants had increased levels of stress during the pandemic, which presented as difficulties with sleep as well as changes in feeding and mood/fussiness. Given infant mental health is impacted by caregiver mental health and attachment [28], it is unsurprising that even infants would be affected by a community-wide stressor such as a pandemic. These important findings present an opportunity for intervention. Primary care providers should be especially attuned to sleep and feeding patterns as well as caregiver reports of fussiness in infants during community-wide stressors, and provide targeted education, counseling, and support for caregivers in these areas. This is especially important as these more subtle signs of stress may be less apparent than the more externalizing concerns such as behavioral outbursts from toddlers and preschool-aged children.

Perhaps most significantly, our study included children presenting to a primary care office for routine care. For almost half (44%) of patients experiencing stress, this was not the presenting reason for the visit but was only identified as part of routine preventive care. This provides an opportunity to reach a broader spectrum of children across a community, not just those with high levels of stress or those who would seek care for these concerns. In addition, this setting presents an opportunity for intervention strategies in places where young children are already presenting for routine care and are already familiar to families, as two thirds of patients in the study group were returning patients. Possible prevention or early-intervention strategies could include awareness and education for primary care providers, which supports ongoing work in the United States to strengthen training for pediatricians around mental health care [2,3,12,14]. Stress in young children may be related to increased stress in the caregiver [8,16,29], whether through a change in parenting habits or a change to the parent–child relationship. Findings from this study suggest that during periods of community-wide stress such as a pandemic, primary care practitioners should provide counseling to caregivers of young children on the importance of positive parenting and healthy attachment, reinforce healthy age-appropriate sleep routines, and offer support and/or resources to caregivers for managing their own stress.

There were several limitations to this study. To overcome selection bias which can occur in studies of cross-sectional surveys, as well as minimize the impact of caregiver perception of behaviors, our study design relied on routine provider documentation instead of validated screening questionnaires. However, as our five outcome measure domains are part of routine care in the United States various providers may view and document these issues differently (e.g., some providers may view sleep regression in infancy as a common problem and therefore provide minimal documentation about it). This could have potentially led to an underestimation of prevalence. We also noted a significant difference between study groups in terms of insurance type, with a larger percentage of our study group on public insurance. This could have impacted prevalence rates of stress in this group. However, as above, we felt this difference was more indicative of the changes our patient panel experienced during this time and was reflective of trends seen across our entire health system, and less of an indication of an influx of higher risk patients during the study period. We also saw an increase in tele-health visits during the study period, which was consistent with practice changes during this time. However, our study design focused on the presence of a concern (e.g., “parent has concerns about child’s sleep”), not the degree to which that concern was documented; therefore, prevalence counts should not have been impacted by visit type. In addition, for the limited time wellness visits were conducted over tele-health in our practice, the same Bright Futures [23] documentation templates were used. Finally, our study population was limited to a single geographical area in the United States. However, our health system does also care for patients from surrounding states, and given our location in Washington DC our clinic cares for families from diverse backgrounds, ethnicities, and nationalities.

Based on our findings, we suggest several areas for future study. As this is one of few studies of emotional health impacts of the global pandemic on infants and young children under 6 years, further study of this age group is needed in other communities to better characterize the impact of a community-wide stressor on very young children. Longitudinal or cohort studies should be conducted to better understand if this emotional distress is time-limited or continues beyond the acute stressor. In addition, longitudinal studies are needed to evaluate for any long-term impacts of pandemic-related stress on childhood development, healthy attachment, or on mental health later in childhood. Further research is also needed to test prevention and early-intervention strategies such as positive parenting interventions, especially in primary care settings.

## 5. Conclusions

This is one of very few studies of children under 6 years during the COVID-19 pandemic, including infants. Even very young children and infants were not spared from the stress of the global pandemic. These findings have particular significance given that early childhood is a critical time for developing foundations of emotional health as well as for the development of the growing brain. Stress in young children is often related to increased stress in the caregiver, which can impact not only parenting habits but also the relationship between the parent and child. Signs of emotional stress in our study population were identified in a primary care office during routine care. This highlights a unique opportunity for early intervention and/or prevention in settings where young children are already presenting for routine care (e.g., visits for growth and monitoring, immunization administration, etc.). Proper awareness and education for those who provide routine care to young children across communities, such as pediatricians, GP’s, or even community health workers, can be an important resource to provide counseling for caregivers to support the emotional health of young children during large community-wide stressors.

## Figures and Tables

**Table 1:** Patient demographics.

	Control (%)	Study (%)	*p*-Value
Gender			*p* = 0.057
Male	96 (48%)	115 (57.5%)	
Female	104 (52%)	85 (42.5%)	
Age			*p* = 0.117
0–12 months	111 (55.5%)	106 (53%)	
13–36 months	55 (27.5%)	51 (25.5%)	
36–71 months	34 (17%)	43 (21.55)	
Insurance			*p* = 0.003
Private	181 (90.5%)	160 (80%)	
Public	19 (9.5%)	40 (20%)	
First Encounter			*p* = 0.675
Established patient	128 (64%)	132 (66%)	
New patient	72 (36%)	68 (34%)	
Visit Type Where Concern Raised			*p* < 0.001
Tele-health	1 (0.5%)	22 (11%)	
In-Person	78 (39%)	58 (29%)	
N/A	121 (60.5%)	120 (60%)	

**Table 2:** Prevalence of stress presenting by domain in each cohort.

	Control (%)	Study (%)	*p*-Value
Concern by Domain			
Sleep	13 (6.5%)	28 (14%)	*p* = 0.013
Behavior	9 (4.5%)	15 (7.5%)	*p* = 0.207
Mood	11 (5.5%)	15 (7.5%)	*p* = 0.417
Feeding	43 (21.5%)	50 (25%)	*p* = 0.407
Toileting	30 (15%)	23 (11.5%)	*p* = 0.302

**Table 3:** Multivariable logistic regression adjusting for age, gender, and insurance type.

			*p*-Value	Odds Ratio
Sleep Concerns	NO	YES	*p* = 0.009	2.56 (CI 1.27–5.15)
0–12 months	88	18 (64.3%)		Reference
13–36 months	43	8 (28.6%)	*p* = 0.742	0.88 (CI 0.41–1.88)
36–71 months	41	2 (7.14%)	*p* = 0.158	0.48 (CI 0.18–1.33)
Behavior Concerns	NO	YES	*p* = 0.371	1.5 (CI 0.62–3.65)
0–12 months	104	2 (13.3%)		Reference
13–36 months	44	7 (46.7%)	*p* < 0.001	13 (CI 2.82–60.4)
36–71 months	37	6 (40%)	*p* < 0.001	18.5 (CI 4.0–86)
Mood	NO	YES	*p* = 0.241	1.65 (CI 0.72–3.79)
0–12 months	93	13 (86.7%)		Reference
13–36 months	49	2 (13.3%)	*p* = 0.011	0.15 (CI 0.03–0.65)
36–71 months	43	0 (0%)		
Feeding	NO	YES	*p* = 0.473	1.2 (CI 0.73–1.95)
0–12 months	72	34 (68%)		Reference
13–36 months	39	12 (24%)	*p* = 0.038	0.55 (CI 0.31–0.97)
36–71 months	39	4 (8%)	*p* < 0.001	0.15 (CI 0.06–0.4)
Toileting	NO	YES	*p* = 0.3	0.73 (CI 0.4–1.32)
0–12 months	94	12 (52.2%)		Reference
13–36 months	42	9 (39.13%)	*p* = 0.641	0.85 (CI 0.43–1.67)
36–71 months	41	2 (8.7%)	*p* = 0.117	0.48 (CI 0.19–1.2)
Referral to Specialist				
Private insurance				Reference
Public insurance			*p* = 0.017	2.62 (CI 1.19–5.81)

## Data Availability

The data presented in this study is not publicly available due to data protection regulations of our Institutional Review Board but are available on request from the corresponding author.
